# Crystal structure of the *Schizosaccharomyces pombe* U7BR E2-binding region in complex with Ubc7

**DOI:** 10.1107/S2053230X19009786

**Published:** 2019-08-02

**Authors:** Zachary S. Hann, Meredith B. Metzger, Allan M. Weissman, Christopher D. Lima

**Affiliations:** a Structural Biology Program, Sloan Kettering Institute, 1275 York Avenue, New York, NY 10065, USA; b Tri-Institutional Training Program in Chemical Biology, Memorial Sloan Kettering Cancer Center, New York, NY 10065, USA; c Laboratory of Protein Dynamics and Signaling, Center for Cancer Research, National Cancer Institute, National Institutes of Health, Frederick, MD 21702, USA; d Howard Hughes Medical Institute, 1275 York Avenue, New York, NY 10065, USA

**Keywords:** endoplasmic reticulum, ERAD, ubiquitin, protein degradation, ubiquitin-conjugating enzyme, E2, E2-binding protein, *Schizosaccharomyces pombe*

## Abstract

Endoplasmic reticulum (ER)-associated degradation (ERAD) is a protein quality-control pathway in eukaryotes in which misfolded ER proteins are polyubiquitylated, extracted and ultimately degraded by the proteasome. This process involves ER membrane-embedded ubiquitin E2 and E3 enzymes, as well as a soluble E2 enzyme (Ubc7 in *Saccharomyces cerevisiae* and UBE2G2 in mammals). E2-binding regions (E2BRs) that recruit these soluble ERAD E2s to the ER have been identified in humans and *S. cerevisiae*, and structures of E2–E2BR complexes from both species have been determined. In addition to sequence and structural differences between the human and *S. cerevisiae* E2BRs, the binding of E2BRs also elicits different biochemical outcomes with respect to E2 charging by E1 and E2 discharge. Here, the *Schizosaccharomyces pombe* E2BR was identified and purified with Ubc7 to resolve a 1.7 Å resolution co-crystal structure of the E2BR in complex with Ubc7. The *S. pombe* E2BR binds to the back side of the E2 as an α-helix and, while differences exist, it exhibits greater similarity to the human E2BR. Structure-based sequence alignments reveal differences and conserved elements among these species. Structural comparisons and biochemistry reveal that the *S. pombe* E2BR presents a steric impediment to E1 binding and inhibits E1-mediated charging, respectively.

## Abstract 2

A 1.7 Å resolution co-crystal structure of U7BR from *Schizosaccharomyces pombe* in complex with Ubc7 is reported. Structural comparisons and biochemistry suggest that U7BR presents a steric impediment to E1 binding and inhibits E1-mediated charging, respectively.

## 1. Introduction   

Eukaryotic membrane and secreted proteins are translated on and folded in the endoplasmic reticulum (ER; Smith *et al.*, 2011 ▸). Misfolding of this class of proteins elicits ER stress and can affect cellular homeostasis. The accumulation of such misfolded proteins and ER stress underlies many human disorders (Guerriero & Brodsky, 2012 ▸). Cells protect against this through pathways that include ER-associated degradation (ERAD), a process in which incorrectly folded proteins are ubiquitylated, marking them for extraction and subsequent degradation by the proteasome (Zattas & Hochstrasser, 2015 ▸).

ERAD requires specific E2 and E3 enzymes, including a conserved soluble E2: UBE2G2 (humans) or Ubc7 (*Saccharomyces cerevisiae*) (Smith *et al.*, 2011 ▸; Ye & Rape, 2009 ▸; Hiller *et al.*, 1996 ▸; Biederer *et al.*, 1996 ▸; Tiwari & Weissman, 2001 ▸). These E2s are recruited to the ER by membrane-embedded proteins. In humans, this is accomplished by gp78, a polytopic E3 in which the cytoplasmic tail includes a RING domain that confers E3 activity, a CUE domain that binds Ub noncovalently (Biederer *et al.*, 1997 ▸; Prag *et al.*, 2003 ▸) and a C-terminal 27-residue UBE2G2-binding region (G2BR; Chen *et al.*, 2006 ▸). X-ray crystallo­graphic studies of the UBE2G2–G2BR complex show G2BR to bind to the back side of the E2 (the face opposite the catalytic cysteine) as an α-helix (Li *et al.*, 2009 ▸; Das *et al.*, 2009 ▸). In isolation, G2BR decreases the rate of UBE2G2~Ub charging by the Ub-activating E1 enzyme but increases the rate of RING domain-dependent UBE2G2~Ub discharge to substrates (Das *et al.*, 2009 ▸).

In *S. cerevisiae*, Ubc7 is recruited to the ER by Cue1, a single-pass transmembrane protein that includes a transmembrane helix, a CUE domain and a Ubc7-binding region (U7BR) composed of 53 residues at its C-terminal end (Kostova *et al.*, 2009 ▸). Unlike gp78 in humans, *S. cerevisiae* Cue1 does not possess a RING domain or catalyze E3 activity, but it enhances the rate of Lys48-linked Ub chain building on ERAD substrates (Bazirgan & Hampton, 2008 ▸; Kostova *et al.*, 2009 ▸) by pairing with one of two ER-embedded RING domain-containing Ub E3s: Hrd1 and Doa10 (Biederer *et al.*, 1997 ▸; Ravid *et al.*, 2006 ▸; Swanson *et al.*, 2001 ▸). The U7BR domain is necessary for the degradation of Ubc7 substrates, presumably because it recruits Ubc7 to the ER (Kostova *et al.*, 2009 ▸). Unlike human G2BR, Cue1 and its U7BR domain increase the rate of E1-mediated E2 charging (Metzger *et al.*, 2013 ▸).

Human and *S. cerevisiae* E2BRs both bind to the back side of their respective E2s through α-helices, but share less than 65% sequence similarity, and only two E2–E2BR hydrogen-bond contacts appear to be conserved in their structures. In addition, while human G2BR is composed of a single long α-helix, *S. cerevisiae* U7BR consists of a shorter α-helix that is flanked by two α-helices (H1 and H3) and a 3_10_-helix (g1) (Li *et al.*, 2009 ▸; Das *et al.*, 2009 ▸; Metzger *et al.*, 2013 ▸).

The differences between the human and *S. cerevisiae* E2/E2BR systems suggest that the analysis of other ERAD E2/E2BRs could reveal additional differences that contribute to E2 binding and recruitment in other organisms. Here, we identify the putative E2BR domain in *S. pombe* Cue4 and present a 1.7 Å resolution co-crystal structure of this E2BR in complex with *S. pombe* Ubc7. Structure-based comparisons with human and *S. cerevisiae* E2BRs reveal overall similarities in addition to structural features that are distinct from either *Homo sapiens* G2BR or *S. cerevisiae* U7BR. Similar to human G2BR, *S. pombe* U7BR may inhibit E1-mediated E2 charging.

## 2. Materials and methods   

### 2.1. Cloning   

The *S. pombe* Ubc7 gene was inserted into the pET-29b+ vector using the NdeI and XhoI restriction sites. A thrombin cleavage consensus site (LVPRGS) was inserted between the C-terminus of Ubc7 and the C-terminal His_6_ tag. Residues 152–215 of *S. pombe* Cue4 (U7BR) were inserted into the pET-28b vector using the NdeI and XhoI restriction sites, yielding a construct with an N-terminal thrombin-cleavable His_6_ tag.

### 2.2. Protein expression and purification   

*S. pombe* Ubc7 and U7BR were expressed in *E. coli* BL21-CodonPlus (DE3)-RIL cells. The cells were grown in SuperBroth at 37°C to an OD_600_ of 1.0. IPTG was added to 1 m*M* and the temperature was reduced to 30°C. 4 h after induction, the cells were centrifuged for 12 min at 4°C and 5000*g*, suspended in buffer consisting of 20 m*M* Tris pH 8, 350 m*M* NaCl, 20% sucrose, flash-frozen in liquid nitrogen and stored at −80°C until lysis. Cells were lysed by sonication and cleared by centrifugation at 40 000*g* and 4°C for 30 min. The cleared lysate was applied onto Ni–NTA Superflow resin (Qiagen), washed in 20 m*M* Tris pH 8.0, 350 m*M* NaCl, 1 m*M* β-mercaptoethanol, 20 m*M* imidazole and eluted in the same buffer with 250 m*M* imidazole.

### 2.3. Preparation of the Ubc7–U7BR complex   

A solution containing *S. pombe* Ubc7 and U7BR each at 16 µ*M* was incubated for 80 min on ice in a buffer consisting of 20 m*M* Tris pH 8.0, 50 m*M* NaCl, 1 m*M* TCEP. The solution was subjected to size-exclusion chromatography on a HiLoad 26/600 Superdex 75 prep-grade column (GE) equilibrated in 20 m*M* Tris pH 8.0, 50 m*M* NaCl, 1 m*M* TCEP. Fractions containing the Ubc7–U7BR complex were pooled, concentrated, flash-frozen in liquid nitrogen and stored at −80°C until further use.

### 2.4. Crystallization and data collection   

The *S. pombe* Ubc7–U7BR complex was subjected to sparse-matrix crystallization screening using the sitting-drop vapor-diffusion method with a Mosquito robot (100 nl well solution added to 100 nl protein solution). The Classics Suite and the PEGs Suite from Qiagen were tested at protein-complex concentrations of 3.3, 6.6 and 10 mg ml^−1^ and temperatures of 4 and 18°C. An initial crystallization hit appeared within 24 h using 6.6 mg ml^−1^ Ubc7–U7BR, 100 m*M* Tris pH 8.5, 200 m*M* MgCl_2_, 30% PEG 4000 at 18°C. Refinement resulted in crystallization conditions that consisted of 100 m*M* Tris pH 8.0, 200 m*M* MgCl_2_, 30% PEG 4000 (Table 1 ▸). 1 µl of this well solution was added to 1 µl protein solution composed of 6.6 mg ml^−1^ Ubc7–U7BR, 20 m*M* Tris pH 8.0, 50 m*M* NaCl, 1 m*M* TCEP on a cover slide and set over 500 µl well solution in a hanging-drop vapor-diffusion experiment. The initial crystals diffracted to 2.9 Å resolution; although α-helical density was observed on the back side of Ubc7, the maps were of insufficient quality to unambiguously determine the register of U7BR. The single crystal used to collect the reported data was harvested from a crystallization experiment at 18°C after approximately one year. The crystal was cryoprotected by stepwise transfer to drops containing mother liquor plus increasing concentrations of MPD up to a final concentration of 20% before being flash-cooled in liquid nitrogen. Data were collected on the NE-CAT beamline 24-ID-C at the Advanced Photon Source (APS), Argonne, Illinois, USA (Table 2 ▸).

### 2.5. Structure determination and refinement   

The data set was indexed, integrated and scaled using *HKL*-2000 (Otwinowski & Minor, 1997 ▸). Data were truncated at 1.7 Å resolution to maintain completeness as the crystal-to-detector distance was not adjusted to obtain higher resolution data for this crystal. *Phaser* (McCoy *et al.*, 2007 ▸) was used for molecular replacement using Ubc7 from the *S. cerevisiae* Ubc7–U7BR structure (PDB entry 4jqu; Metzger *et al.*, 2013 ▸) as a search model (with the U7BR chain deleted). The initial solution revealed electron density consistent with a helix near the back side of Ubc7 in a similar position to that observed for the human and *S. cerevisiae* ERAD E2BRs. A polyalanine model was initially placed into this density and refinement resulted in density characteristic of amino-acid side chains. The sequence of *S. pombe* Cue4 was built into this density. Coordinates were refined via iterative rounds of refinement and rebuilding using *PHENIX* (Adams *et al.*, 2010 ▸) and *Coot* (Emsley *et al.*, 2010 ▸) (Table 3 ▸).

### 2.6. Transthiolation assay   

*S. pombe* Uba1^Δ1–12^ and Ub were purified as described previously (Olsen & Lima, 2013 ▸). Reactions contained 10 n*M* Uba1, 500 n*M* Ubc7, 3 µ*M* Ub, 0 or 1 µ*M* U7BR, 10 m*M* MgCl_2_, 20 m*M* HEPES pH 7.5, 50 m*M* NaCl, 0.1% Tween-20. 2 m*M* ATP was used to start the reaction. Reactions were run at room temperature and quenched after the indicated time by mixing the reaction with an equal volume of 4× LDS NuPAGE loading dye (Life Technologies). Lanes labeled ‘+ βME’ in Supplementary Fig. S1 contained 358 m*M* β-mercapto­ethanol after mixing the sample and dye. Reactions were resolved by 4–12% Bis-Tris SDS–PAGE with MOPS running buffer (Life Technologies), stained with SYPRO Ruby (Bio-Rad) and imaged on a Typhoon FLA 9500. Ubc7 bands were quantified using *ImageJ* (NIH). Each data point in Fig. 4(*a*) is the average of three independent experiments, and error bars represent one standard deviation. Values were normalized to the percentage of E2-SH remaining by dividing the values by the average value at the zero time point and multiplying by 100.

## 3. Results and discussion   

### 3.1. Identification, preparation and crystallization of the *S. pombe* Ubc7–U7BR complex   

Protein sequences for *S. pombe* Cue4 U7BR were aligned and domain boundaries were estimated based on the known structure of *S. cerevisiae* U7BR in complex with Ubc7. Alignments between residues 152–215 of *S. pombe* Cue4 and residues 151–203 of *S. cerevisiae* Cue1 revealed that the two sequences share 66% similarity and 38% identity within the predicted E2-binding surface. The E2 proteins are better conserved than their respective E2BRs, with the *S. pombe* and *S. cerevisiae* Ubc7 sequences sharing 75% similarity and 61% identity. For comparison, the sequences of *S. pombe* U7BR and *H. sapiens* G2BR (residues 579–600 of gp78) share 70% similarity and 47% identity, while the sequences of *S. pombe* Ubc7 and human UBE2G2 share 83% similarity and 68% identity.

Full-length *S. pombe* Ubc7 and U7BR encompassing residues 152–215 of Cue4 were expressed in *E. coli* and purified. *S. pombe* Ubc7 and U7BR were incubated in a stoichiometric ratio and purified by size-exclusion chromatography. The peak was pooled, concentrated and subjected to sparse-matrix crystallization screening. After refining the crystallization conditions, a crystal was grown that diffracted to 1.7 Å resolution in space group *P*2_1_2_1_2_1_ with a single complex in the asymmetric unit (Tables 1 ▸ and 2 ▸). Phases were determined by molecular replacement using the coordinates of *S. cerevisiae* Ubc7 (without U7BR) as a search model. The preliminary structure revealed helical densities on the back side of Ubc7, and U7BR from *S. pombe* was manually built into this density based on unambiguous densities for the respective side chains (Table 3 ▸).

### 3.2. Comparison of ERAD E2–E2BR complexes from *H. sapiens*, *S. cerevisiae* and *S. pombe*   

**Figure 1:**
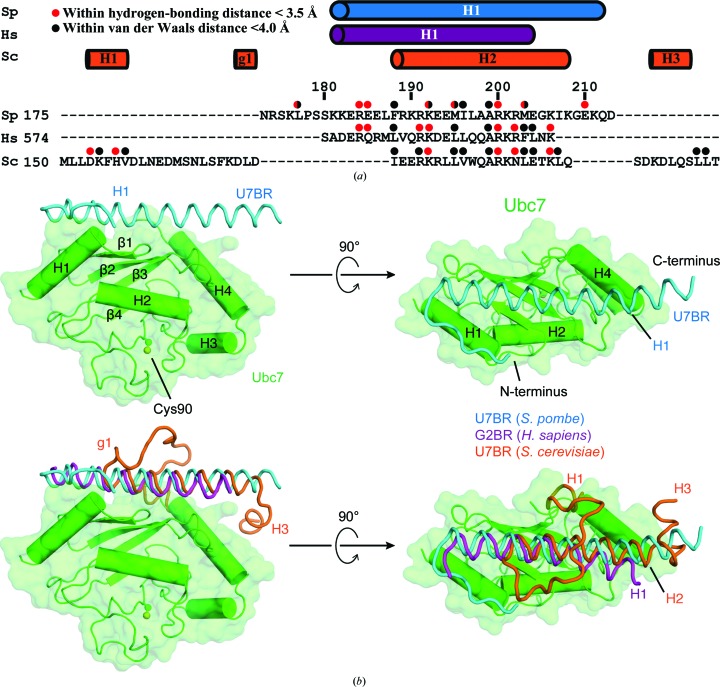
Crystal structure of the *S. pombe* Ubc7–U7BR complex. (*a*) Structure-based alignment of *S. pombe* U7BR, *H. sapiens* G2BR and *S. cerevisiae* U7BR. Residues within helices are represented by cylinders above the alignment. Residues that interact with Ubc7 are labeled with red or black circles. (*b*) *S. pombe* Ubc7 shown as a green cartoon and surface with U7BR represented as a cyan ribbon. Cys90 of Ubc7 is shown as sticks and spheres, and the N- and C-termini of U7BR are labeled. Below, similar structures of the E2BRs of *H. sapiens* (purple; PDB entry 3h8k; Das *et al.*, 2009 ▸) and *S. cerevisiae* (orange; PDB entry 4jqu) superimposed. Figures were made with *PyMOL* (v.2.0; Schrödinger).

*S. pombe* U7BR binds to the back side of Ubc7 as a long α-helix comprising residues 180–213 [Figs. 1 ▸(*a*) and 1 ▸(*b*)] in a manner that resembles E2 binding by *H. sapiens* G2BR and *S. cerevisiae* U7BR (Li *et al.*, 2009 ▸; Das *et al.*, 2009 ▸; Metzger *et al.*, 2013 ▸). The E2-binding helices (*S. pombe* U7BR helix H1, *H. sapiens* G2BR helix H1 and *S. cerevisiae* U7BR helix H2) align with respect to the E2 between residues 188 and 204 of *S. pombe* U7BR [residues 173 and 189 of *S. cerevisiae* U7BR; residues 582 and 598 of *H. sapiens* G2BR; Fig. 1 ▸(*b*)]. At the C-terminal end of human G2BR, the remainder of helix H1 (residues 599–60) bends towards the β2β3 loop of UBE2G2. The C-terminal ends of the two yeast U7BR helices continue to align until residue 208 of *S. pombe* U7BR (residue 193 of *S. cerevisiae* U7BR). The C-terminal end of *S. pombe* U7BR continues as a helix until residue 213, making it the longest contiguous helix of the three E2BRs. After residue 193, *S. cerevisiae* U7BR bends towards helix H4 of Ubc7, forming a separate α-helix, H3, composed of residues 196–200. At the N-terminal end of the helix, *S. cerevisiae* U7BR (residues 150–172) turns towards the H2 face of Ubc7, forming a short 3_10_-helix (g1), before looping over its own E2-binding H2 helix to form an N-terminal H1 α-helix. In contrast, the N-termini of *S. pombe* U7BR and *H. sapiens* G2BR have longer E2-binding helices that extend over the H1 helix of the E2. Human G2BR and *S. pombe* U7BR diverge at residue 180 of *S. pombe* U7BR (residue 574 of G2BR), where human G2BR ends and *S. pombe* U7BR turns towards the H2 face of Ubc7, forming a seven-residue N-terminal extension that lies on top of the H1 helix of Ubc7 and is unique among the three E2BRs. While the *H. sapiens* and *S. cerevisiae* E2BR domains used for crystallization were mostly ordered, with only a few residues at the N-terminus of G2BR that did not have sufficient density to be modeled, the longer *S. pombe* U7BR construct used for crystallization had 22 N-terminal residues that were not observed in electron density (Li *et al.*, 2009 ▸; Das *et al.*, 2009 ▸; Metzger *et al.*, 2013 ▸). The *S. pombe* Ubc7–U7BR complex buries a surface area of 2423 Å^2^ between the E2 and U7BR, as calculated using *CNS* (Brunger, 2007 ▸). For comparison, the E2–E2BR complexes from *S. cerevisiae* and *H. sapiens* bury surface areas of 2076 and 1947 Å^2^ between the E2 and the E2BR, respectively.

### 3.3. Ubc7–U7BR intermolecular contacts   

**Figure 2:**
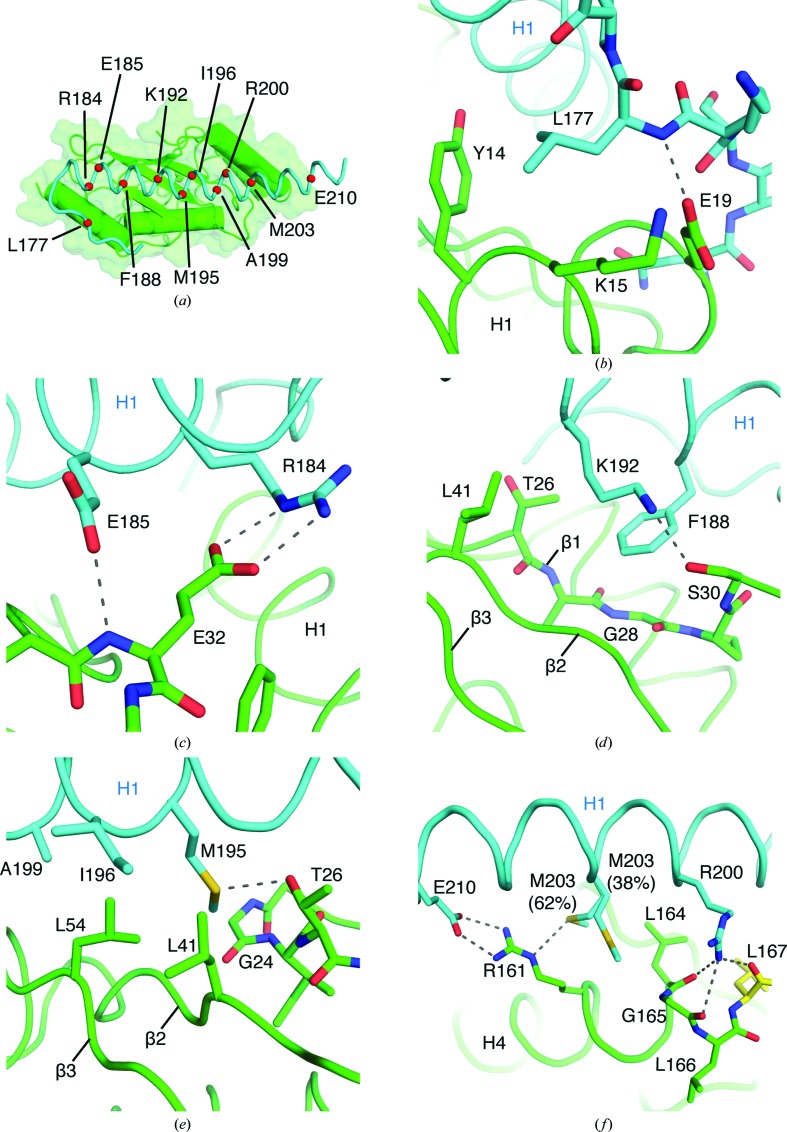
Intermolecular contacts between Ubc7 and U7BR. (*a*) *S. pombe* Ubc7–U7BR complex with α carbons of U7BR residues in contact with Ubc7 depicted as red spheres. (*b*) Interaction between Leu177 of U7BR (cyan) and Tyr14, Lys15 and Glu19 of Ubc7 (green). (*c*) Interaction between Arg184 and Glu185 of U7BR (cyan) and Glu32 of Ubc7 (green). (*d*) Interaction between Phe188 and Lys192 of U7BR (cyan) and Thr26–Ser30 and Leu41 of Ubc7 (green). (*e*) Interaction between Ala199, Lys192, Met195 and Ile196 of U7BR (cyan) and Gly24, Thr26, Leu41 and Leu54 of Ubc7 (green). (*f*) Interaction between Arg200, Met203 and Glu210 of U7BR (cyan) and Arg161 and Leu164–Leu167 of Ubc7 (green and yellow). Residues in yellow are part of a thrombin cleavage site artifact at the C-terminus of Ubc7.

11 U7BR residues have main-chain or side-chain atoms within hydrogen-bonding or van der Waals distance of Ubc7 [Fig. 2 ▸(*a*)]. While similarities exist with respect to intermolecular contacts within the *H. sapiens*, *S. cerevisiae* and *S. pombe* E2–E2BR complexes, many of the contacts discussed here are unique to the *S. pombe* system. In the N-terminal portion of *S. pombe* U7BR, the backbone amide N atom of Leu177 of U7BR is 2.8 Å from a side-chain carboxylate O atom of Glu19 in Ubc7, to which it may establish a hydrogen bond [Fig. 2 ▸(*b*)]. The δ1 carbon of the Leu177 side chain is within van der Waals distance of the ∊ and ζ carbons of Tyr14 in the H1 helix of Ubc7, and its δ2 carbon is 3.7 Å from the α and β carbons of Lys15 in the same helix, possibly forming hydrophobic interactions.

The N-terminal end of the H1 helix of U7BR approaches the β1β2 loop of Ubc7. In this interface, the ∊ and η nitrogensof Arg184 are within hydrogen-bonding distance of the ∊ oxygen of the side-chain carboxylate of Glu32 [Fig. 2 ▸(*c*)]. The main-chain amide N atom of Glu32 is 2.8 Å from a side-chain carboxylate oxygen of Glu185 in U7BR, potentially creating an additional hydrogen bond.

The side chain of Phe188 of U7BR contributes to a hydrophobic interface with Ubc7; the ∊ and ζ carbons are 4.0 and 3.7 Å from the α carbon of Gly28 in the β1 strand of Ubc7, respectively [Fig. 2 ▸(*d*)]. A δ carbon of Phe188 is 3.9 Å from the main-chain α carbon of Ser30 in Ubc7; on the opposite side of the aromatic ring of Phe188, an ∊ carbon is 3.4 Å from the side-chain γ2 carbon of Thr26. Nearby, the ζ nitrogen in the side chain of Lys192 is 3.0 Å from the side-chain hydroxyl O atom of Ser30. In addition, the α, β and γ carbons of Lys192 are 3.9 Å from a side-chain δ carbon in Leu41 in the β2 strand of Ubc7, and the γ carbon of Lys192 is 4.0 Å from the γ2 carbon in the side chain of Thr26 of Ubc7, possibly contributing hydrophobic contacts within the Ubc7–U7BR interface.

Additional contacts are established by Met195 and Ile196 of U7BR [Fig. 2 ▸(*e*)], with interactions between the side-chain δ sulfur of Met195 and the side-chain hydroxyl O atom of Thr26 in Ubc7, which are 3.1 Å apart. The side-chain methyl carbon of Met195 is 3.8 Å from one of the δ carbons of Leu54 in the β3 strand of Ubc7 and 4.0 Å from the α carbon of Gly24 in the β1 strand of Ubc7. The main-chain α carbon of Ile196 is 3.7 Å from the side chain of Leu54 in Ubc7, and the side chain of Ile196 is within van der Waals distance of Leu41 and Leu54.

Leu54 in Ubc7 also appears to contribute hydrophobic contacts to the side chain of Ala199 in U7BR; the β and δ2 carbons of Leu54 are 3.9 Å from the side-chain β carbon of Ala199 [Fig. 2 ▸(*e*)]. Adjacent to Ala199, one of the η nitrogens of Arg200 is 3.1 Å from the main-chain carbonyl oxygen of Leu164 and 3.4 Å from the main-chain carbonyl oxygen of Gly165 at the C-terminus of Ubc7 in the H4 helix [Fig. 2 ▸(*f*)]. The same N atom in the side chain of Arg200 is 2.9 Å from the main-chain carbonyl oxygen of Leu167, a nonphysiological contact as the E2 was expressed with a C-terminal thrombin-cleavable His tag and the final residue of *S. pombe* is Leu166.

The C-terminal end of U7BR includes Met203, a residue whose S atom is within hydrogen-bonding distance of the side-chain ∊ nitrogen of Arg161 in the H4 helix of Ubc7 [Fig. 2 ▸(*f*)]. Met203 is also within van der Waals distance of Ubc7: the ∊ carbon is 3.9 Å from the side-chain δ carbon of Leu164 in the H4 helix of Ubc7. The side-chain β carbon of Met203 is 3.8 Å from the same δ carbon of Leu164. In an alternate side-chain conformation of Met203 (38% occupancy), the side-chain methyl carbon is within van der Waals distance of the α carbon of Gly165 and the γ carbon of Arg161 in Ubc7. In Glu210 of U7BR, the two side-chain carboxylate O atoms are 2.7 and 2.9 Å from the two η nitrogens of Arg161 in helix H4 of Ubc7, potentially forming a salt bridge.

### 3.4. Interactions that are conserved between *H. sapiens*, *S. cerevisiae* and *S. pombe*   

**Figure 3:**
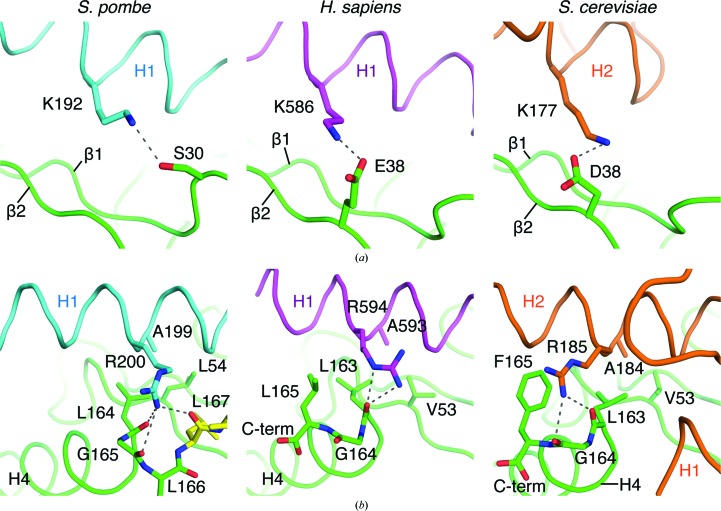
Conserved E2-interacting residues among ERAD E2BRs. (*a*) A conserved E2BR lysine may form a hydrogen bond to a Ubc7 side chain in *S. pombe* (cyan), *H. sapiens* (purple) and *S. cerevisiae* (orange). (*b*) A conserved alanine and arginine interact with residues of Ubc7 in *S. pombe* (cyan), *H. sapiens* (purple) and *S. cerevisiae* (orange).

The Ubc7–U7BR structures from *S. cerevisiae* and *S. pombe* were aligned with the UBE2G2–G2BR structure from *H. sapiens* (Das *et al.*, 2009 ▸; Metzger *et al.*, 2013 ▸) [Figs. 1 ▸(*a*) and 1 ▸(*b*)]. Three *S. pombe* U7BR residues that interact with Ubc7 are structurally and genetically conserved in the two other species: Lys192, Ala199 and Arg200. In *S. pombe* U7BR, this lysine is within hydrogen-bonding distance of Ser30 in the β1β2 loop of Ubc7. At the same E2BR position in *S. cerevisiae*, the side chain of Lys177 of U7BR is within hydrogen-bonding distance of the side chain of Asp38 on the β2 strand of Ubc7 [Fig. 3 ▸(*a*)]. Similarly, in the *H. sapiens* structure the side chain of Lys586 of G2BR is within hydrogen-bonding distance of the side chain of Glu38 on the β2 strand of UBE2G2. In the position corresponding to Asp38 of *S. cerevisiae* Ubc7 and Glu38 of *H. sapiens* UBE2G2, *S. pombe* Ubc7 has Asp39, but it is more than 3.5 Å from the ζ nitrogen of Lys192 on U7BR. In the *H. sapiens* and *S. cerevisiae* structures, the β1β2 loops of the E2s are too far from the conserved lysine to form a hydrogen bond.

Ala199 and Arg200 of *S. pombe* U7BR are also conserved; they correspond to Ala593 and Arg594 in *H. sapiens* G2BR and Ala184 and Arg185 in *S. cerevisiae* U7BR, respectively. Ala199 is within van der Waals distance of Leu54 on the β3 strand of Ubc7 in the *S. pombe* structure. Similarly, Ala593 of G2BR is within van der Waals distance of Val53 on the β3 strand of UBE2G2 and Ala184 is within van der Waals distance of Val53 on the β3 strand of *S. cerevisiae* Ubc7 (Das *et al.*, 2009 ▸; Metzger *et al.*, 2013 ▸) [Fig. 3 ▸(*b*)]. This may represent a conserved hydrophobic interaction. The interaction between the side chain of Arg200 in *S. pombe* U7BR and main-chain carbonyl oxygens at the C-terminal end of Ubc7 may also be conserved. In *H. sapiens*, the ∊ nitrogen and one of the η nitrogens of Arg594 in G2BR are within hydrogen-bonding distance of the main-chain carbonyl oxygen of Leu163 in UBE2G2, and in the *S. cerevisiae* structure an η nitrogen of Arg185 appears to interact with the carbonyl oxygens of Leu163 and Gly164 near the C-terminus of Ubc7 [Fig. 3 ▸(*b*)].

### 3.5. E1–Ubc7 transthiolation in *S. pombe*   

**Figure 4:**
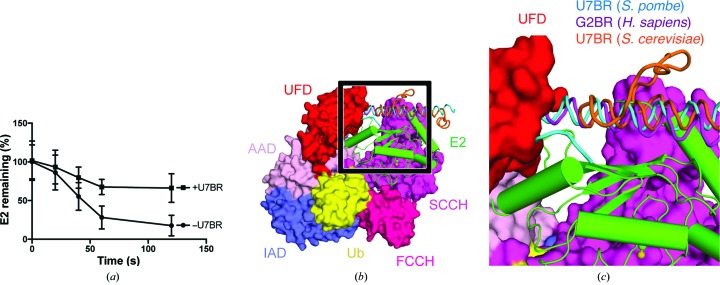
*S. pombe* U7BR impedes Ubc7 transthiolation. (*a*) Percentage of free Ubc7 remaining in a transthiolation assay with and without excess U7BR. Error bars represent one standard deviation. (*b*) E2–E2BR co-crystal structures from *S. pombe* (PDB entry 6op8), *H. sapiens* (PDB entry 3h8k) and *S. cerevisiae* (PDB entry 4jqu; Metzger *et al.*, 2013 ▸) were modeled into the crystal structure of *S. pombe* E1–Ubc4–Ub–ATP (PDB entry 4ii2; Olsen & Lima, 2013 ▸) by superposing the E2 structures. The black square indicates the area displayed in (*c*). (*c*) The N-terminal portions of *S. pombe* U7BR and *H. sapiens* G2BR, but not *S. cerevisiae* U7BR, clash with the UFD domain of E1. IAD, inactive adenylation domain; AAD, active adenylation domain; Ub, ubiquitin; UFD, ubiquitin-fold domain; FCCH, first catalytic cysteine half-domain; SCCH, second catalytic cysteine half-domain.

*S. pombe* U7BR decreases the rate of Ubc7~Ub charging *in vitro*, similar to the effect of G2BR on UBE2G2~Ub charging in *H. sapiens* (Fig. 4 ▸; Das *et al.*, 2009 ▸). Superposition of E2–E2BR crystal structures on the structure of a cross-linked Ub E1–E2 complex reveals potential steric clashes between the N-termini of *H. sapiens* G2BR or *S. pombe* U7BR and the Ub-fold domain (UFD) of the Ub E1 (Olsen & Lima, 2013 ▸). In contrast, because the Ubc7-binding H2 helix of *S. cerevisiae* U7BR does not extend as far in the N-terminal direction, it does not cover the E2 surface contacted by the UFD. This may explain why *S. cerevisiae* U7BR is able to stimulate transthiolation *in vitro*, rather than repressing it as in the other two species (Metzger *et al.*, 2013 ▸).

While *S. pombe* U7BR appears to interfere with E1–Ubc7 binding, it presents no obvious structural impediment to Ubc7–E3 RING domain binding. When the E2 proteins from the structures of *S. pombe* Ubc7–U7BR and human UBE2G2 bound to the RING and G2BR domains of gp78 were superposed, U7BR did not preclude interactions with the RING domain-binding surface of the E2 (Das *et al.*, 2013 ▸). This is consistent with the observation that E2BRs from both *H. sapiens* and *S. cerevisiae* enhance the E3-mediated ubiquitylation activity and RING domain-binding affinity of ERAD-associated E2s (Das *et al.*, 2009 ▸; Metzger *et al.*, 2013 ▸).

## 4. Discussion   

Recruitment of a soluble E2 (Ubc7 in yeast and UBE2G2 in humans) to the endoplasmic reticulum is essential for many ubiquitylation events in ERAD (Zattas & Hochstrasser, 2015 ▸). Although this core function is conserved, it is interesting that the E2-binding regions (E2BRs) have such a varied sequence and structure. In addition, isolated U7BR from *S. cerevisiae* increases the rate of E1–E2 transthiolation, suggesting that Cue1 may stimulate transthiolation at the ER (Metzger *et al.*, 2013 ▸). In contrast, the ERAD E2BRs from *H. sapiens* and *S. pombe* appear to have the opposite effect: the E2BR inhibits E1-mediated E2 charging [Fig. 4 ▸(*a*) and Supplementary Fig. S1] (Das *et al.*, 2009 ▸). This observation suggests that E2s are charged with Ub prior to ER recruitment. A structural basis for the opposing effects observed for E1–E2 transthiolation can be rationalized by superposing E2–E2BR structures onto Ub E1–E2 complexes. These models reveal that the N-terminal ends of E2BRs from human and *S. pombe* present a steric clash with the E2-binding Ub-fold domain (UFD) of E1 [Figs. 4 ▸(*b*) and 4 ▸(*c*)]. In contrast, the *S. cerevisiae* U7BR does not occlude the E2-binding surface of the UFD. *S. pombe* appears to be more similar to mammals than to *S. cerevisiae* with respect to the regulation of heat-shock factors and some aspects of lipid metabolism; it is possible that the recruitment of soluble ERAD E2s to the ER may be another process in which *S. pombe* is a closer uni­cellular model organism than *S. cerevisiae* (Raychaudhuri *et al.*, 2012 ▸; Gallo *et al.*, 1991 ▸; Hoffman *et al.*, 2015 ▸).

This work identified the *S. pombe* U7BR and presents an atomic resolution model of a Ubc7–U7BR complex. While the E2-binding mode of *S. pombe* U7BR is globally similar to those observed in the human and *S. cerevisiae* systems, their biochemical effects and interactions include elements that are unique among the three ERAD E2–E2BR structures solved to date (Metzger *et al.*, 2013 ▸; Das *et al.*, 2009 ▸, 2013 ▸). The similarities and differences in E2–E2BR complexes solved thus far may aid in the identification of additional E2BRs in other systems.

## Supplementary Material

PDB reference: Ubc7–U7BR complex, 6op8

Supplementary Figure S1. DOI: 10.1107/S2053230X19009786/pg5083sup1.pdf

## Figures and Tables

**Table 1:** Crystallization

Method	Hanging drop
Plate type	VDXm plate with sealant
Temperature (K)	291
Protein concentration (mg ml^−1^)	6.6
Buffer composition of protein solution	20 m*M* Tris pH 8.0, 50 m*M* NaCl, 1 m*M* TCEP
Composition of reservoir solution	100 m*M* Tris pH 8.0, 200 m*M* MgCl_2_, 30% PEG 4000
Volume and ratio of drop	1 µl, 1:1
Volume of reservoir (µl)	500

**Table 2:** Data collection Statistics were calculated using *PHENIX*; values in parentheses are for the outer shell.

Diffraction source	24-ID-C, APS
Wavelength (Å)	0.97910
Temperature (K)	100
Detector	PILATUS 6M-F
Crystal-to-detector distance (mm)	250
Rotation range per image (°)	0.2
Total rotation range (°)	180
Exposure time per image (s)	0.2
Space group	*P*2_1_2_1_2_1_
*a*, *b*, *c* (Å)	26.72, 79.05, 94.52
α, β, γ (°)	90, 90, 90
Resolution range (Å)	47.26–1.703 (1.764–1.703)
Completeness (%)	99.39 (98.12)
Total No. of reflections	129954 (12467)
No. of unique reflections	22673 (2195)
Wilson *B* factor (Å^2^)	14.49
Multiplicity	5.7 (5.7)
*R*_merge_ (%)	5.62 (22.32)
CC_1/2_ (%)	99.9 (97)
CC* (%)	100 (99.2)
〈*I*/σ(*I*)〉	25.94 (9.05)

**Table 3:** Structure refinement Statistics were calculated using *PHENIX*; values in parentheses are for the outer shell.

Resolution range (Å)	47.26–1.703 (1.764–1.703)
σ Cutoff	*F* > 1.380σ(*F*)
No. of reflections (work/free)	22633 (2187)/1169 (98)
*R*_work_/*R*_free_ (%)	16.80 (19.69)/19.76 (24.65)
No. of non-H atoms	
Total	1952
Protein	1732
Ligand	6
Solvent	214
R.m.s. deviations	
Bonds (Å)	0.010
Angles (°)	1.04
Average *B* factors (Å^2^)	
Overall	20.50
Protein	19.54
Ligand	34.27
Water	27.88
Statistics from *MolProbity*	
Ramachandran favored† (%)	97.09 [200]
Ramachandran outliers† (%)	0 [0]
Clashscore	96th percentile
*MolProbity* score	91st percentile
PDB code	6op8

† The number of residues is given in brackets.
