# Supplementary material for: Crystal structure of the Schizosaccharomyces pombe U7BR E2-binding region in complex with Ubc7
Source: Acta Crystallogr F Struct Biol Commun. 2019 Aug 2;75(Pt 8):552–60. doi: 10.1107/S2053230X19009786 (PMC6688661; doi:10.1107/S2053230X19009786)
Supplement: Supplementary file 1 [file f-75-00552-sup1.pdf]

STRUCTURAL BIOLOGY COMMUNICATIONS

**Volume 75 (2019)**

**Supporting information for article:**

# Crystal structure of the *Saccharomyces pombe* U7BR E2-binding domain in complex with Ubc7

**Zachary S. Hann, Meredith B. Metzger, Allan M. Weissman and Christopher D. Lima**

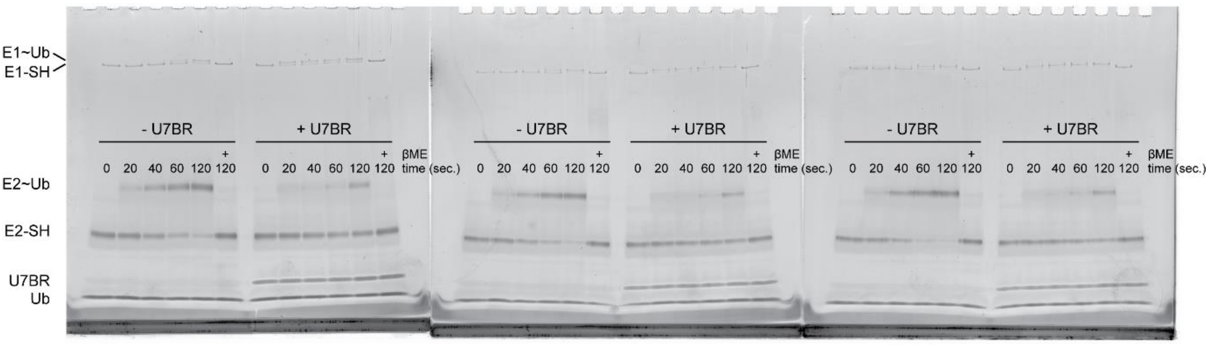


**Figure S1** SDS-PAGE analysis of transthiolation assays. Analysis of E1-mediated transthiolation reactions as measured by the appearance of Ubc7~Ub over the course of two minutes. SDS-PAGE gels were stained using SYPRO Ruby. Each reaction (with or without U7BR) was conducted in triplicate. The 120-second time point was re-run in a separate lane after treatment with β-mercaptoethanol (βME) to disrupt the thioester bond. The 0 minute time point was taken before addition of ATP. Densitometry of E2-SH bands are plotted in Fig. 4*a*.
